# Effects of the Interaction Between Time-on-Task and Task Load on Response Lapses

**DOI:** 10.3390/bs14111086

**Published:** 2024-11-13

**Authors:** Jingqiang Li, Yanru Zhou, Tianci Hao

**Affiliations:** 1 Safety Science and Engineering College, Civil Aviation University of China, No. 2898 Jinbei Highway Dongli District, Tianjin 300300, China; 2 The Key Laboratory of Road and Traffic Engineering, Ministry of Education, Shanghai 201804, China; 3 School of Transportation Engineering, Tongji University, Shanghai 201804, China

**Keywords:** time-on-task, task load, response lapses, fNIRS

## Abstract

To investigate the interaction effects of prolonged working periods and different task loads on response lapses, focusing on the mechanisms of delayed responses and error lapses. Professionals such as pilots, truck drivers, and nurses often face extended work hours and fluctuating task loads. While these factors individually affect performance, their interaction and its impact on response lapses remain unclear. Twenty participants completed the Uchida–Kraepelin (U–K) Psychological Test and a dual-task version with functional near-infrared spectroscopy. Independent variables were time-on-task and task load. Dependent variables included measures of fatigue, arousal, workload, task performance (delayed and error rates), and brain functional connectivity. Both time-on-task and task load significantly affected cerebral connectivity, response lapses, workload (frustration level), fatigue, and arousal. Arousal levels significantly decreased and reaction times increased after 60 min of work. Cognitive resource regulation became challenging after 90 min under high task load levels. A decline in the connection between the prefrontal and occipital cortex during high-load tasks was observed. The findings provide insight into the mechanisms of response lapses under different task load levels and can inform strategies to mitigate these lapses during extended work periods. This study’s findings can be applied to improve work schedules and fatigue management in industries like aviation, transportation, and healthcare, helping reduce response lapses and errors during extended work periods under high task load conditions.

## 1. Introduction

**Figure 1:**
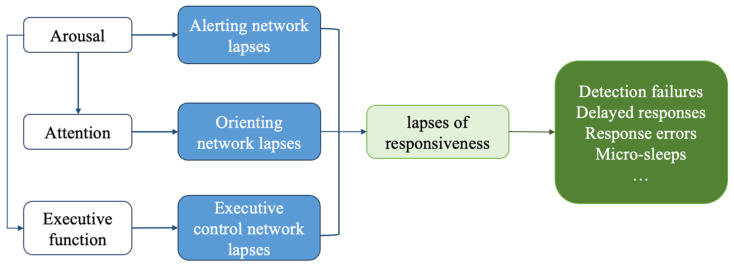
The psychological mechanism theory and performances of response lapses (adapted from [10,11]).

Professionals such as pilots, truck drivers, and nurses often experience prolonged working periods and variable task load, which can lead to response lapses and thereby increase the risk of accidents. Response lapses are disruptions in sensory and motor skills or cognitive performance that typically last between 0.5 and 15 s and manifest as detection failures, delayed responses, response errors, and micro-sleeps [1]. Lapses are caused by an imbalance between available cognitive resources and the requirements of the task. Cognitive resources are sensitive to arousal levels and fatigue [2,3]. As Figure 1 shows, any problem in any link of this network may lead to a lapse [4,5]. There are two theories that explain response lapses. First, the mindlessness theory suggests that response lapses are due to unconscious shifts or withdrawal of attention [6]. For example, lapses are more likely to occur in monotonous and automated tasks [7]. Second, the resource depletion theory suggests that cognitive resources are limited. As cognitive resources available for attention decrease, they become depleted, leading to lapses [8]. Buckley et al. found that lapses of attention were more likely to occur in high-load tasks, whereas micro-sleeps occurred in low-complexity tracking tasks [9].

Time-on-task (TOT) is the length of time spent actively involved in a task [12]. Task load is the objective demands associated with the task, whereas workload reflects the subjective experiences of these demands, including mental, physical, and temporal aspects. The same level of task load may lead to different workload levels among individuals, depending on their skills, expertise, or personal characteristics [13]. In practice, task load and TOT often interact to affect fatigue [14]. For example, the cruise phase of a flight is typically monotonous, has a low task load, and has a long duration [15]. The takeoff and landing phases impose a higher task load, as a large amount of information is processed in a short period and quick decisions and responses are needed [16,17]. Therefore, when exploring the physiological and psychological mechanisms of different types of lapses, TOT and task load must be considered. Moreover, traditional human-operated tasks, such as vehicular operation, are incrementally shifting toward roles that necessitate continuous supervisory control. This paradigmatic shift, while potentially enhancing systemic efficiency and operational safety, simultaneously introduces novel challenges to the cognitive performance and safety of workers.

Current research on TOT and task load spans a variety of approaches and applications, emphasizing the complexity of these factors and their effects on cognitive performance. Studies have shown that increased task difficulty can lead to a higher workload level, resulting in decreased task performance [18,19]. Li et al. evaluated around 20 min of simulated flight performance of pilots under low, medium, and high task loads [20]. The results indicate that participants on a high task load had higher scores for the National Aeronautics and Space Administration Task Load Index (NASA-TLX), heart rate, and prefrontal cortex activation. However, there is currently limited research on response lapses in continuous low-load tasks, such as in air traffic control [21], truck driving [22], aviation [15], and nursing [23], in which there are frequently periods of sustained labor surpassing one hour. Numerous research tasks have durations of less than 1 h [24,25]. There is a particular need for additional research on low-task-load work over long periods of time.

The assessment of fatigue, workload, and lapses has traditionally involved a triangulation of subjective self-reports, behavioral evaluations, and physiological metrics [13]. Existing studies have shown that several measures are sensitive to lapses; among them are dual-task methods [26], the Psychomotor Vigilance Test (PVT) [27], and the Uchida–Kraepelin Psychological Test (U–K test) [28]. The PVT paradigm classifies a reaction time > 500 ms as a lapse [27], but lapses manifest through various behaviors. Therefore, it is necessary to design optimized experiments to investigate the mechanisms underlying the lapses (delayed response and error). In the realm of neuroergonomics, researchers are employing a diverse set of metrics to understand and quantify workload and fatigue; yet, some integration into real-world occupational settings remains impractical [29]. Functional near-infrared spectroscopy (fNIRS) emerges as a non-invasive cerebral monitoring modality, serving as an efficacious instrument for assessing the functional connectivity within cortical networks [30]. The fNIRS has been used in both simulated and actual flight contexts to appraise workload [31,32]. Meanwhile, there are several scales sensitive to fatigue, arousal, and workload, including the Karolinska Sleepiness Scale (KSS) [33] and Stanford Sleepiness Scale (SSS) [34] for measuring sleepiness, Activation-Deactivation Adjective Check List (AD-ACL) [35] for evaluating arousal levels, and NASA-TLX [36] for workload assessment. However, some past studies have shown a discrepancy between self-perception and objective results [37]. Therefore, it is necessary to analyze and discuss the consistency between subjective and objective results and the reliability of common scales.

A comprehensive investigation into the interplay between TOT and task load is imperative to understand and mitigate response lapses in high-stake environments. This study aims to investigate the interaction of TOT and task load on lapses, with an emphasis on their physiological and psychological underpinnings. Such insights are vital for developing advanced monitoring systems, risk alert technologies, and effective interventions to minimize human error among control personnel.

In this study, the U–K test was used to classify response lapses into errors and delayed responses. The mechanisms underlying lapses were further explored by combining the U–K test with fNIRS, as well as the subjective assessment of fatigue, arousal, and workload levels. We hypothesized the following:

**H1:**  *The rate of delayed responses will increase more in single-task conditions than in dual-task conditions with the growth of TOT, while high task load will result in a higher error rate compared to low task load.*

**H2:**  *TOT and workload have a significant effect on cerebral functional connectivity within and between brain regions.*

**H3:**  *The fatigue levels (as measured by KSS and SSS) will increase more in dual-task conditions compared to single-task conditions, while arousal levels (as measured by AD-ACL) will decrease more in single-task than in dual-task conditions.*

**H4:**  *Subjective measures (KSS, SSS, AD-ACL, NASA-TLX) significantly correlate with behavior (as measured by total number, delayed responses, and errors per 10 min) and cerebral functional connectivity (within and between brain regions).*

This investigation is intended to synthesize a patterned understanding of the behavioral and cognitive dynamics of lapses under varied TOT and task load conditions. Based on the findings, we propose scientific and targeted recommendations for preventing and treating response lapses.

## 2. Methods

### 2.1. Participants

In this study, using a priori power analysis to calculate the total sample size of the within-factors ANOVA repeated measures, given power = 0.80, α = 0.05, and effect size = 0.28, the required sample size was N = 20 [16]. To mitigate potential data quality issues, 22 university students with good aviation knowledge were recruited by putting up posters on the campus. After the experiment, each participant received 200 RMB as a reward. During the U–K test, one participant’s data showed partial record loss due to an E-prime 3.0 software issue, while another participant’s fNIRS data indicated a 20 min period of poor signal quality. These two participants were removed, leaving 20 participants with valid data, 10 men and 10 women, aged 21–24 years (23.29 ± 0.81).

All participants were right-handed and in good health, with normal or corrected-to-normal visual acuity and no history of psychiatric or sleep disorders. Participants had a Pittsburgh Sleep Quality Index score of less than 5 and were required to get adequate sleep (≥7 h) in the 24 h period before the experiment and maintain a sleep diary. Prior to the experiment, all participants voluntarily provided informed consent and were notified of the experimental procedure. The study was conducted in accordance with the Declaration of Helsinki and approved by the Ethics Committee of the Research Institute of Civil Aviation Safety (CAUC-PSY-2022-04-02, 30 April 2022).

### 2.2. Measures

This study included both objective and subjective measures. The dependent variables comprised KSS, SSS, and AD-ACL scores; NASA-TLX values; total number of responses every 10 min (TN/10 min); delayed response rate and error rate; and brain functional connectivity strength, measured using functional connectivity in and between brain regions.

#### 2.2.1. Objective Measures

##### Behavioral Response Test

The U–K test was developed by Japanese clinical psychologist Yuzabur Uchida and is based on the “continuous addition method” proposed by German psychologist and psychiatrist Emil Kraepelin [38]. The U–K test, a low workload task, is widely used to evaluate ability and mental performance [39] and in pilot selection and training. The UK test requires participants to complete a sequential addition task in a specific table. Starting with the first row, participants are asked to add two adjacent numbers and fill in the single digits of the sum in a specified place. The test also requires participants to concentrate for a long time and quickly and accurately perform calculations. The test has the same level of difficulty for well-educated participants, is easily accessible, has low practice effects, eliminates the effects of different difficulty and familiarity levels of difficulty and familiarity to some extent, and can effectively respond to individual ability, sustained attention, personality, and behavior.

In this study, the U–K test was automated using E-Prime 3.0. An addition problem using digits up to ten would appear on the screen, and participants would enter the correct digit using the keyboard (numeric nine-box grid). The next question would appear immediately, and the system recorded response time (RT) and accuracy (AC) for each question. In the dual-task experiment with a high workload, radiotelephony communication was selected as the second task. Participants were required to recite radiotelephony communication after hearing the instructions, and their performance was influenced by their attention allocation, short-term memory, thinking, and language skills. The audio material selected for this study was a classic land–air call of moderate command length, consisting of letters–numbers–words–numbers, for approximately 7 s each. E-Prime 3.0 was used for automatic random playback, and the audio information and time points played were recorded.

##### Functional Near-Infrared Spectroscopy

**Figure 2:**
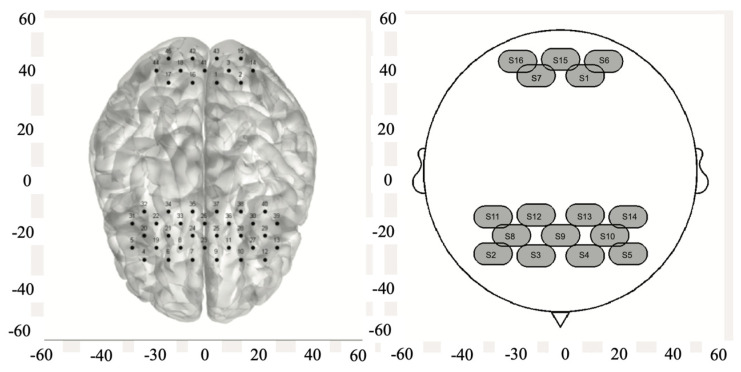
Prefrontal and occipital cortical channels.

The data were collected in the frontal and occipital cortex. Research has found that the occipital region, domicile to the visual cortex, engages in extensive neural dialog with the prefrontal and temporal lobes—networks imperative for executing routine tasks [40,41]. Furthermore, the prefrontal lobe is instrumental for sophisticated cognitive operations [42]. Data on cerebral blood oxygen were acquired using Nir Smart (Danyang Huichuang Medical Equipment Co. Ltd., Beijing, China) based solely on the standard long-distance channels. The brain regions examined in this study were the prefrontal and occipital cortices, which were located using a 3D localizer [43] and based on the international 10/20 system. The laser diodes used in this experiment had wavelengths of 730 and 850 nm and a sampling frequency of 11 Hz. The device could detect real-time changes in HbO_2_ concentration using 16 light-source transmitter sites, and 16 signal receiver sites for 45 channels in total were placed in the right prefrontal cortex (RPFC), left prefrontal cortex (LPFC), right occipital lobe (ROL) and left occipital lobe (LOL) (see Figure 2).

The left figure shows 45 channels, with 32 located in the frontal lobe and 13 in the occipital lobe. The right figure displays the distribution of light source emitter points, with 11 located in the frontal lobe and 5 in the occipital lobe.

#### 2.2.2. Subjective Measures

This study utilized the KSS and SSS, which are widely used to measure fatigue [33,44]. The KSS is a 9-point self-reported scale ranging from 1 (extremely alert) to 9 (extremely sleepy; fighting sleep). The SSS is a 7-point self-reported scale, ranging from 1 (feeling active and vital; alert; wide awake) to 7 (almost in reverie; sleep onset soon; lost struggle to remain awake) to indicate fatigue level. The AD-ACL was used to measure participants’ subjective arousal states at four levels: general activation (GA), deactivation sleep (DS), high activation (HA), and general deactivation (GD), with higher GA/DS values indicating higher arousal levels [35].

Workload was assessed using the NASA-TLX with six dimensions: mental demand, physical demand, time demand, operational performance, effort, and frustration [45]. Each question has a maximum score of 100, with a higher score indicating a more demanding workload. In this study, we used raw scores for each dimension without applying any weighting, and the results were analyzed separately for each dimension.

### 2.3. Design and Procedure

**Figure 3:**
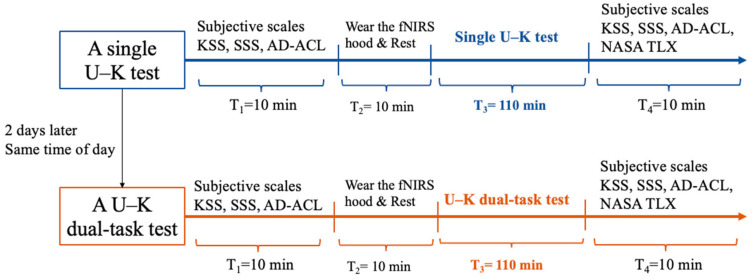
Experimental protocol.

A within-subject, repeated measures experimental design was employed to explore the combined effect that workload and TOT have on response lapses (Figure 3). Each participant completed two experiments with two workload levels wearing the fNIRS cap. To reduce the interference of circadian rhythm, each participant underwent a second experiment (U–K dual-task) at the same time of day, two days after the first experiment (U–K single test).

**Figure 4:**
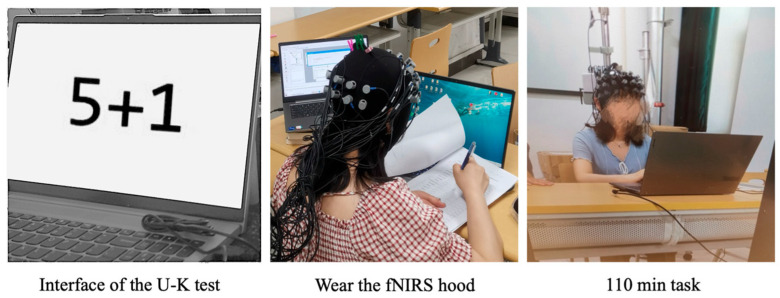
Uchida–Kraepelin test interface.

Before the experiment, participants practiced the U–K test until they were familiar with the task requirements; the experimental interface is shown in Figure 4. Before the experiment, participants practiced the single-task U–K test and dual-task test for each of 20 min until they successfully completed at least three trials with a consistent performance criterion, defined as no statistically significant differences in reaction time and accuracy across the trials, ensuring they were familiar with the task requirements. The laboratory’s ambient temperature was controlled at 24 ± 1 °C, and the illumination level was set to 70–100 Lux [46,47].

### 2.4. Data Analysis

#### 2.4.1. Data Preprocessing

##### U–K Tests Data

Data are presented as mean (M) ± standard deviation (σ), and the single and dual-task U–K tests are denoted by corner symbols 1 and 2, respectively. The U–K test behavioral data were preprocessed, and the RT data were confirmed to follow a normal distribution using the Shapiro–Wilk test. Abnormal RTs, accounting for approximately 0.87% of the total data, were removed based on the Chauvenet criterion [48]. The This U–K test explored behavioral performance by the TN/10 min of questions participants completed in 10 min and number of response lapses, where the response lapses included delayed responses and errors. The mean RT in the U–K test (M RT = 1254.6 ms) and standard deviation (σ = 823.2 ms) were calculated, and the 3724.2 ms (M RT + 3σ) was considered the threshold for delayed response lapses [49].

##### fNIRS Data

Functional connectivity (FC) indicates brain activity and dynamic synchronization of neural activity signals in different encephalic regions. Spline interpolation was used to identify and eliminate unreliable motions, setting a standard deviation threshold of six and a peak threshold of 0.5. Physiological noise, including cardiac and respiratory activities, was filtered from 0.021 to 0.145 Hz, and the path difference factor was set to −6–6 [50].

In fNIRS research, oxygenated hemoglobin (HbO_2_) is preferred over deoxygenated hemoglobin (HbR) because HbO_2_ typically exhibits larger signal changes during brain activation, leading to a higher signal-to-noise ratio [51,52]. These changes are more directly associated with neural activity, as increased blood flow delivers more oxygen-rich blood to active regions. Conversely, HbR variations are more complex and susceptible to noise, making HbO_2_ a more reliable measure. Therefore, this study only analyzed HbO_2_ levels. The “Network” module of NirSpark 1.7.5 software extracted HbO_2_ concentrations at the sampling points. Further, the Pearson correlation coefficients of HbO_2_ concentrations in each channel of the time series were analyzed, and Fisher Z-transformed values were used to determine the strength of the connections [53].

##### Subjective Measures Data

We subtracted the subjective measurement values of KSS, SSS, and AD-ACL after the experiment from the measurements before the experiment for each participant. Subsequently, we calculated the change score in subjective assessment results and used Δ to represent it.

#### 2.4.2. Statistical Analysis

Statistical analysis was performed using SPSS 26.0. Two levels of task load (single and dual-task) and TOT (10 levels: {1: = (0–10 min), 2: = (10–20 min), 3: = (20–30 min), 4: = (30–40 min), 5: = (40–50 min), 6: = (50–60 min), 7: = (60–70 min), 8: = (70–80 min), 9: = (90–100 min), 10: = (100–110 min)}) were used as within-subject variables. A two-way repeated-measures ANOVA was chosen to analyze the effects and interactions of task load and TOT on behavioral performances and brain FC strength, and the Greenhouse–Geisser correction was selected to estimate sphericity (*p* < 0.05), using the ε correction coefficient to correct for the degrees of freedom. Simple effects tests were conducted when the interactions were significant and main effects tests when they were not, along with post hoc pairwise comparisons [54].

A Wilcoxon test was used to compare KSS, SSS, and AD-ACL scores before and after the task (ΔKSS, ΔSSS, and ΔGA/DS) to explore the effect task load and 2 h task had on fatigue and arousal. NASA-TLX was used to evaluate the subjective workload of the single- and dual-task tests. Spearman’s correlation test was used to explore the correlations between subjective and objective performance, testing the consistency of subjective measures with objective behaviors and physiological results.

## 3. Results

### 3.1. Uchida–Kraepelin Performance Test

In the repeated-measures ANOVA, task load and time-on-task showed no statistically significant main effects or interaction on error rate and delayed response rate, as shown in Table 1. Specifically, for task load, the F-values were 3.767 (*p* = 0.067, η^2^ = 0.165) for error rate and 1.334 (*p* = 0.57, η^2^ = 0.017) for delayed response rate. For time-on-task, the F-values were 3.084 (*p* = 0.09, η^2^ = 0.14) for error rate and 1.814 (*p* = 0.487, η^2^ = 0.041) for delayed response rate, with adjusted degrees of freedom. The interaction between task load and time-on-task also showed no significant effects, with F-values of 2.825 (*p* = 0.102, η^2^ = 0.129) for error rate and 1.985 (*p* = 0.136, η^2^ = 0.095) for delayed response rate. These results indicate that neither task load nor time-on-task had a statistically significant impact on performance measures. As for averages for TN/10 min, task load (F = 0.348, *p* = 0.562, η^2^ = 0.016) and TOT (F = 1.532, *p* = 0.239, η^2^ = 0.561) had non-significant main effects but a significant interaction effect (F = 5.498, *p* = 0.004, η^2^ = 0.821).

**Figure 5:**
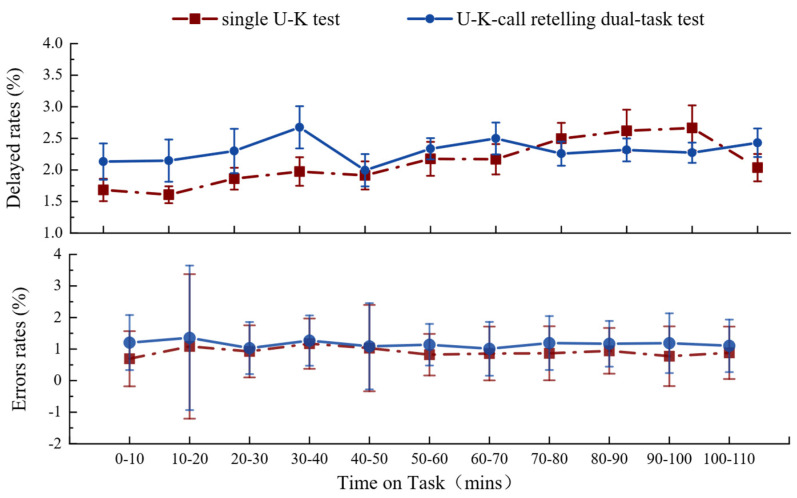
Delayed response and error rates for two-task load levels.

Analysis of the delayed response and error rates per 10 min for 2 levels of task load showed that, overall, the rate of lapses in the delayed response was approximately twice as high as the rate of errors (Figure 5). The delayed response rate tended to increase slowly over time for different task load levels. The delayed response rate increased from 0 to 100 min in the single U–K test, whereas the dual-task delayed response rate increased significantly in the first half of the task (1–40 min), peaking at 30–40 min (2.6%). The delayed response rate was higher in the single-task test than in the dual-task test at 70–100 min. The error rate was relatively stable, without significant fluctuations, with the single- and dual-task error rates at 0.9% and 1.2%, respectively.

**Figure 6:**
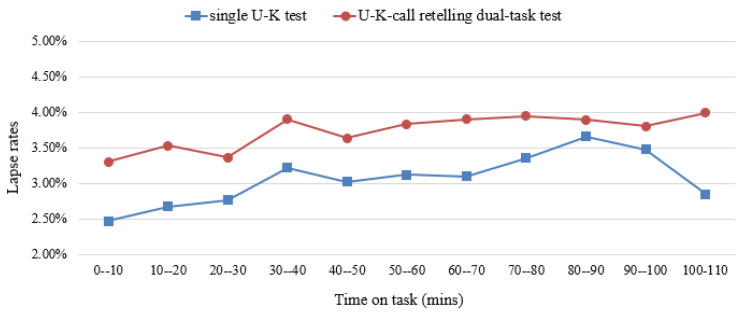
Response lapse rates for two-task load levels.

Figure 6 compares the mean response lapse rates for high and low task load levels. The total lapse rate was higher in the dual-task test than in the single-task test, and attention fluctuated significantly during the behavioral response test. In particular, the number of lapses increased in the first 40 min of the single-task test and started to decrease after reaching the first peak, which may be related to the regulatory mechanism of cognitive flexibility.

The incidence of response lapses increased again after 60 min of continuous work and was the highest at approximately 90 min. In the dual-task test, the lapse rate in the first 50 min followed a trend similar to that of the single-task test; however, after 50 min of the continuous dual-task test, the individual lapse rate increased slowly, rose rapidly in the later part of the task (100–110 min), and peaked at the end of the task. The task was divided into five stages (initiation, hold 1, control 1, hold 2, and control 2) based on observed fluctuations in lapse rate and cognitive performance trends [24].

**Figure 7:**
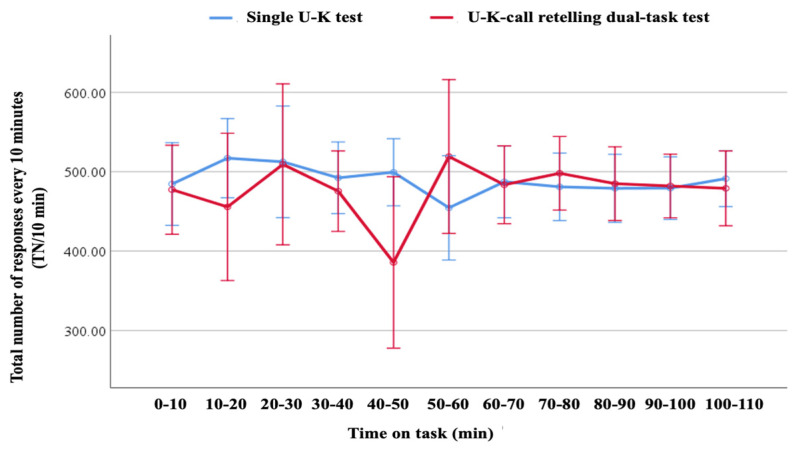
Total number of questions with two task load levels.

Figure 7 shows the averages for TN/10 min. Task load showed a significant simple effect for high- and low-load tasks in 40–50 min (F = 5.773, *p* = 0.026, η^2^ = 0.216), with the TN/10 min in the dual-task test being significantly less than in the single-task test (M_1_-M_2_ = 113.636). TOT showed a significant simple effect in the dual-task test (F = 2.976, *p* = 0.039, η^2^ = 0.713).

This U–K test explored behavioral performance by the total number of responses every 10 min (TN/10 min).

### 3.2. Functional Near-Infrared Spectroscopy

**Figure 8:**
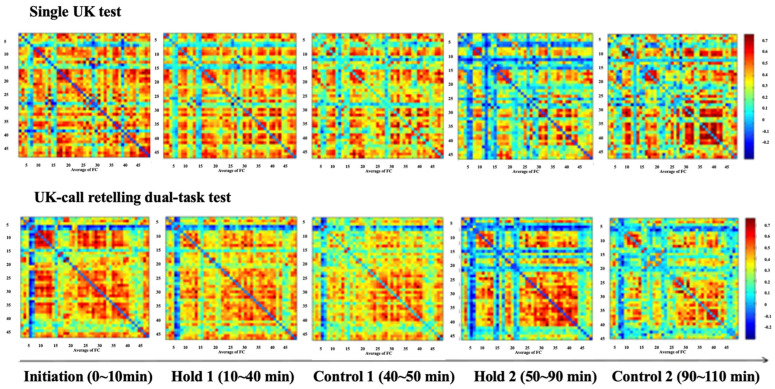
Brain functional connectivity at different TOT points.

The task was divided into five stages: initiation (0–10 min), hold 1 (10–40 min), control 1 (40–50 min), hold 2 (50–90 min), and control 2 (90–110 min), based on fluctuations in behavioral performance. These stages were identified to better capture distinct patterns in behavioral responses rather than using uniform 10 min intervals [24]. Figure 8 shows a heatmap of functional brain connectivity strength, where increased red areas indicate stronger connectivity. The heatmap illustrates how connectivity patterns changed across different task stages and conditions, revealing that in the hold 2 and control 2 phases (after 50 min), participants exhibited significantly lower connectivity, particularly in the dual-task condition. This decrease in connectivity is indicative of reduced neural communication efficiency during prolonged task engagement.

The functional brain connectivity within each channel in different brain regions is shown in Table 2. The main effect of task load on right prefrontal connection strength was statistically significant, with a pairwise comparison showing that the single-task test had a higher connection than the dual-task test (M_1_-M_2_ = 0.077, *p* = 0.004), whereas the hold 1 phase (0.504) was higher than control 1 (0.483, *p* = 0.021) and control 2 (0.452, *p* = 0.019). The main effect of TOT on occipital lobe connections was statistically significant, with pairwise comparisons showing significantly more connection in the initiation (0.497) than in the hold 2 (0.477, *p* = 0.001) and the control 2 (0.409, *p* = 0.000).

The functional brain connectivity between brain regions is shown in Table 3. The main effects of task load and TOT were significant for the strength of the connection between the right and left prefrontal lobes; the interaction effect of task load and TOT was significant. Pairwise comparisons showed that functional brain connectivity in the single-task test was higher than in the dual-task test, except for the initiation, hold 1 (M_1_-M_2_ = 0.058, *p* = 0.001), control 1 (M_1_-M_2_ = 0.082, *p* = 0.000), hold 2 (M_1_-M_2_ = 0.065, *p* = 0.000), and control 2 (M_1_-M_2_ = 0.069, *p* = 0.000) phases. The strength of the connection in the dual-task test increased faster in the initiation and hold 1 phases than in the subsequent phases, and these differences were statistically significant.

The main effect of TOT was significant for the strength of the connection between the right prefrontal and occipital lobes; the interaction effect of task load and TOT was significant. Pairwise comparisons showed that functional brain connectivity was higher in the single-task test than in the dual, except in the initiation phase (*p* < 0.05), and most significant in the hold 2 phase (M_1_-M_2_ = 0.051, *p* = 0.000). In the single- and dual-task tests, the strength of the connection was significantly higher in the initiation and hold 1 phases than in the subsequent phases (*p* < 0.05).

The main effect of TOT was significant for the strength of the connection between the left prefrontal and occipital lobes; the interaction effect of task load and TOT was significant. Pairwise comparisons showed that functional brain connectivity strength was lowest in the control 2 phase in the single-task test and statistically different from the other phases. In the dual-task test, it was significantly higher in initiation and hold 1 than in control 1 and hold 2 (*p* < 0.05).

### 3.3. Subjective Performance

**Figure 9:**
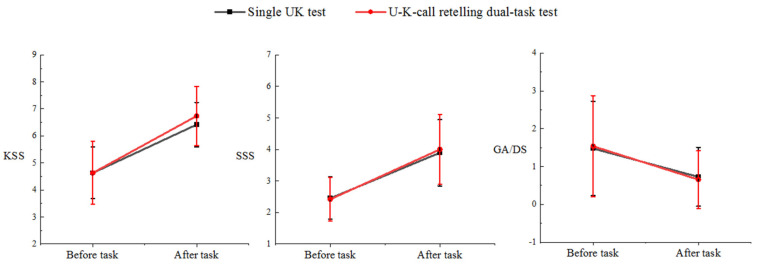
Subjective experience before and after two task load levels.

As illustrated in Figure 9, a series of Wilcoxon tests showed that fatigue significantly increased (KSS_1_: Wilcoxon, N = 20, *p* < 0.000, z = 3.973; KSS_2_: *p* < 0.001, z = 3.866; SSS_1_: *p* < 0.001, z = 3.601; SSS_2_: *p* < 0.001, z = 3.926), while arousal level significantly decreased (GA/DS_1_: *p* = 0.003, z = −3.007; GA/DS_2_: *p* < 0.001, z = −3.945) after the single- and dual-task tests compared to pre-task. Individual fatigue change scores for the dual-task test (ΔKSS = 2.15, ΔSSS = 1.57) were higher than those for the single-task test (ΔKSS = 2.05, ΔSSS = 1.44), whereas arousal change scores were higher for the single-task test (ΔGA/DS = −0.75) than for the dual-task test (ΔGA/DS = −0.69).

**Figure 10:**
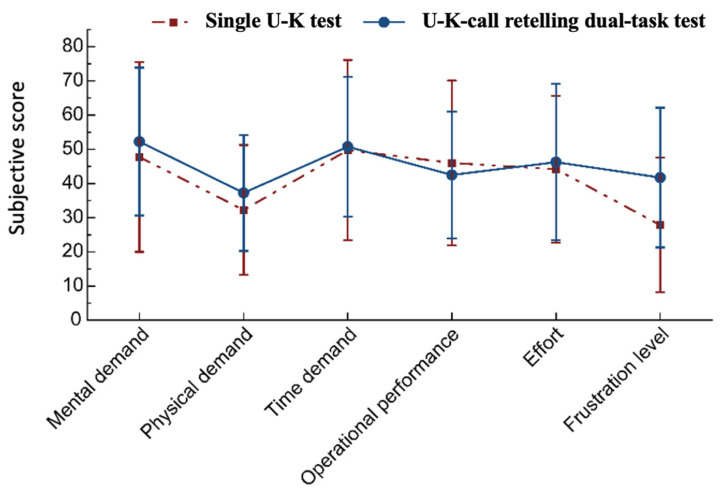
Subjective evaluation of workloads for two task load levels.

As shown in Figure 10, frustration was significantly higher in the dual-task condition compared to the single-task condition (*p* < 0.001, z = 3.419). However, there were no statistically significant differences in other workload components, such as mental and physical demands or effort, between the two conditions. The independent variable in this study was task load (single vs. dual), while the dependent variable was participants’ self-rated workload. These results suggest that although the dual-task condition did lead to a significant increase in frustration, the overall perceived workload across different components did not show substantial differences.

### 3.4. Correlation Between Subjective and Objective Measures

The subjective measures scores were tested for correlations with behavioral performance and brain function connectivity, and the ΔSSS was positively correlated with the number of errors (r = 0.310, *p* = 0.043) and negatively correlated with TN/10 min (Spearman’s *p* = −0.349, *p* = 0.032). The ΔGA/DS was negatively correlated with the number of delayed responses (Spearman’s *p* = −0.731, *p* = 0.048) and positively correlated with the mean connection strength within the right prefrontal cortex (Spearman’s *p* = 0.373, *p* = 0.050).

Correlation analysis of the six dimensions of the NASA-TLX and the mean connection strength during the task revealed that mental demand was negatively correlated with the mean connection strength within the occipital lobe (Spearman’s *p* = −0.421, *p* = 0.026). The lapse rate was negatively correlated with the mean connection strength within the right prefrontal lobe (Spearman’s *p* = −0.454, *p* = 0.015). The frustration level was negatively correlated with the mean connection strength between the right and left prefrontal lobes (Spearman’s *p* = −0.484, *p* = 0.009), between the left prefrontal and occipital lobes (Spearman’s *p* = −0.388, *p* = 0.041), and between the right prefrontal and occipital lobes (Spearman’s *p* = −0.457, *p* = 0.015).

Analysis of the differences in KSS, SSS, and GS/DS scores before and after the task and of the differences in connections in each brain region between the control 2 and initiation phases revealed a positive correlation between the change in GA/DS scores and the mean connection strength within the right prefrontal cortex (Spearman’s *p* = 0.373, *p* = 0.050).

## 4. Discussion

This study investigated the interaction mechanism of TOT and task load on response lapses using a 110 min U–K single test and a U–K dual-task test, fNIRS, and subjective measures. We found that attentional performance showed significant fluctuations at the beginning and end of the task, while the lapse rate increased significantly after 90 min of sustained work, particularly for high-load tasks. The strength of the right prefrontal cortex connection was significantly correlated with subjective perception and attentional performance. Prolonged monotonous tasks led to delayed responses and reduced arousal levels.

### 4.1. Effect of TOT and Task Load on Behavioral Performance

The delayed response rate in the single-task test increased significantly with TOT and surpassed that of the dual-task test at 70–80 min, whereas the error rate decreased, thus validating H1. This result supports the assumption that long work hours reduce arousal levels [2], leading to delayed responses. In contrast, tasks with high load levels, which require more cognitive resources, are more likely to result in errors, validating H1 again. This distinction highlights how different types of task demands can affect performance outcomes. Meanwhile, expanding upon the 30 min experimental framework established by [9], our study elongated the TOT and corroborated similar conclusions. Moreover, during dual-task tests, performance peaked mid-task due to the regulation of cognitive flexibility, but as the task continued, cognitive resources waned, leading to more frequent response lapses [55]. This parallels Gimeno et al. differentiation between active fatigue from mental overload and passive fatigue from under-stimulation in driving scenarios [56].

In this study, the TN/10 min in 40–50 min was significantly lower than that before 40 min. This may be due to an individual’s ability to maintain sustained attention for a certain period, as fatigue occurs at around 40 min and the dual-task test required more cognitive resources [57]. During actual flight operations, either the captain or the first officer may manage the aircraft at different phases, including the first hour after takeoff or approximately an hour before descent, depending on task load distribution and operational procedures [58,59]. These high-load tasks require substantial cognitive resources. According to the regulations in China’s CCAR-93TM-R5, the interval between two shifts for air traffic controllers (2 h) directly engaged in radar control shall not be less than 30 min [60]. Our findings provide a reference for the duration of continuous work shifts, suggesting short breaks every 40 min during high-load tasks to help mitigate fatigue.

### 4.2. Effect of TOT and Task Load on Functional Connectivity of Cortical Brain Networks

The results of this study showed that TOT and task load have significant interactions within brain regions’ cerebral functional connectivity (H2). Studies on brain fatigue using EEGs have also found that alpha and beta wave activity in the frontal brain areas increases with brain load [61]. We found that the right prefrontal lobe is more sensitive to excessive task load levels, such that the right prefrontal cortical state can reflect the load and individual fatigue state to some extent, which is consistent with existing studies [24]. The strength of brain function connections decreased with increasing TOT, resulting in fewer available brain resources and reduced alertness due to participants’ low arousal levels.

The strength of connectivity between different brain networks is influenced by the interaction of task load and TOT, most significantly between the left and right prefrontal cortices, followed by the connectivity between the right prefrontal cortex and the occipital lobe. This supports the findings of studies using EEG where the α and θ coherence of the anterior, middle, and interhemispheric networks is influenced by task load and TOT factors [62]. Generally, increasing cognitive demand tends to lead to an increase in *θ* activity, reflecting greater engagement of attentional and working memory processes [63,64]. However, these changes may vary depending on task characteristics and individual differences. For example, studies have shown that while theta activity often increases with higher cognitive load, α activity might also show task-specific modulation rather than a uniform decline [65]. The connection strength decreased more rapidly with TOT in tasks with a high task load level than in simple tasks. However, the effect of task load on the strength of functional connections was most significant in the control 1 and hold 2 phases (40–90 min), indicating that the level of individual brain fatigue increased significantly during prolonged work (>90 min) regardless of the task load. Therefore, continuous work time should be reasonably controlled, and appropriate breaks should be provided to relieve individuals’ physical and psychological fatigue and enhance their alertness levels [66,67].

### 4.3. Correlation of Subjective Measures with Objective Results

In our study, individual fatigue change scores of dual-task tests were higher than those of the single-task test, whereas arousal change scores were higher for the single-task test than the dual-task test, thus validating H3. Tasks that require continuous vigilance can lead to a significant increase in fatigue, and long and monotonous tasks lead to a significant decrease in arousal levels [9]. The more significant the increase in fatigue and the decrease in arousal levels, and the higher the number of errors and task load, the more accurately individuals can evaluate their performance during a task.

While the KSS assessment showed the expected changes in fatigue levels, indicating its effectiveness, it is important to note that subjective measures may not always align with objective physiological and behavioral performances [24]. For instance, in our study, although participants did not report significant differences in perceived task load across high and low task load conditions, other measures indicated notable changes. Specifically, lapse rates were higher and brain network connectivity strength was lower in the dual-task condition. Moreover, research indicates that individuals may underestimate their fatigue levels, as seen in the findings of [68], where long-haul truck drivers reported lower levels of fatigue compared to airline pilots. This discrepancy emphasizes the need for improved education and training on fatigue awareness among workers to enhance self-evaluation and safety. Establishing a culture of open communication regarding fatigue and errors is crucial for fostering safety in organizations. A Just Culture encourages honest reporting without fear of unjust consequences, promoting better safety practices [69,70].

Connection strength in the right prefrontal lobe was correlated with arousal levels, thus validating H4, providing an objective and reliable physiological indicator and scientific basis for monitoring individual arousal levels. Furthermore, good sleep is a key factor in maintaining optimal arousal levels and, consequently, enhancing task performance. Ensuring sufficient rest before a task can help support sustained attention and cognitive function [71]. Simultaneously, measures are taken to improve the rest environment on board, scientifically rotate rest periods on board, optimize pre-flight preparations and transit procedures, and ensure adequate rest time before missions and during transits. In the NASA-TLX, psychological demands, job performance, and frustration level dimensions were all correlated with brain network connection strength, validating H4 again and further supporting the scientific validity of this scale for workload assessment [72].

### 4.4. Limitations and Future Research

This study has some limitations. First, this study aimed to have participants complete the U–K test on a computer, which differs from the actual work environment; therefore, the conclusions drawn may be somewhat limited. It is hoped that in the future, data from participants’ actual work can be collected to validate and optimize existing findings and conclusions. Second, the participants were right-handed university students; therefore, the conclusions of the study may have limited generalizability in terms of age and different brain dominance dimensions. In future studies, more diverse participants in terms of, for example, age, personality, and brain dominance should be selected. Another limitation is the potential influence of training effects. Since participants repeated the tasks multiple times, practice may have led to learning or adaptation, potentially influencing the results. Future studies could address this by implementing counterbalancing, increasing task complexity, or including a separate group for baseline comparisons to better account for training effects. Consequently, future research could use multi-modal techniques to understand the brain mechanisms during different types of response lapses [73].

## 5. Conclusions

This study compared the patterns of response lapse occurrence in 2 h vigilance tasks under 2 levels of workload using subjective measures, objective behavioral performance, and physiological data. Long and monotonous tasks resulted in lower arousal levels and longer reaction times. This suggests that during the low-workload cruise phase of international flights, crew members may experience arousal lapses, such as delayed responses. While our results do not directly address pre-flight preparations, scheduling, or sleep, optimizing these factors could be beneficial in enhancing pilots’ arousal levels. Task duration significantly affects attentional performance, and we observed performance fluctuations, particularly at the start of a task. After extended periods of continuous work (>90 min), mental fatigue increases significantly, regardless of workload. The takeoff and landing phases, characterized by higher cognitive load, may deplete resources and lead to errors. Therefore, incorporating real-time alertness detection using modern psychological technology could mitigate fatigue. Additionally, strategically adding a second task or stimulus during the cruise phase and controlling task duration during critical phases like takeoff and landing could further address fatigue. These suggestions provide a theoretical basis for effectively reducing response lapses across different flight phases. Furthermore, fNIRS serves as an effective tool for studying the functional connectivity of cortical brain networks, particularly in real-world operating environments such as aviation. Our findings suggest that changes in right prefrontal blood oxygen levels may correlate with individual fatigue and alertness, consistent with existing literature [42].

## 6. Key Points

1. Prolonged work hours combined with high task load levels significantly increase response lapses and fatigue levels in participants.
2. High task load may reduce brain connectivity between the prefrontal and occipital cortices, potentially affecting task performance.
3. Findings suggest that cognitive resource regulation becomes challenging after 90 min of continuous work, requiring targeted interventions.
4. These insights can guide fatigue management strategies to improve safety in industries like aviation, transportation, and healthcare.

## Figures and Tables

**Table 1:** ANOVA results for task load and time-on-task.

	Lapses Type	F(df1, df2)	Greenhouse–Geisser	η^2^
Task load	Error rate	3.767 (1.0, 19.0)	0.067	0.165
	Delayed response rate	1.334 (1.0, 19.0)	0.57	0.017
	TN/10 min	0.348 (1.0, 19.0)	0.562	0.016
Time-on-task	Error rate	3.084 (1.1, 21.1)	0.09	0.14
	Delayed response rate	1.814 (2.9, 54.6)	0.487	0.041
	TN/10 min	1.532 (2.9, 45.6)	0.239	0.561
Task load × Time-on-task	Error rate	2.825 (1.2, 22.2)	0.102	0.129
	Delayed response rate	1.985 (2.6, 49.1)	0.136	0.095
	TN/10 min	5.498 * (2.1, 37.3)	0.004	0.821

* *p* < 0.05.

**Table 2:** Functional connections in brain regions.

	Brain Regions	F(df1, df2)	Greenhouse–Geisser	η2
Task load	Left prefrontal	0.063 (1.0, 17.0)	0.803	0.001
	Right prefrontal	8.895 ** (1.0, 14.5)	0.004	0.114
	Occipital	0.041 (1.3, 16.4)	0.840	0.001
Time-on-task	Left prefrontal	0.746 (2.9, 24.3)	0.553	0.011
	Right prefrontal	2.793 (1.5, 29.2)	0.056	0.039
	Occipital	15.792 *** (2.5, 32.1)	0.000	0.186
Task load × Time-on-task	Left prefrontal	0.391 (3.2, 34.2)	0.783	0.006
	Right prefrontal	1.353 (2.9, 38.5)	0.260	0.019
	Occipital	0.289 (2.9, 43.9)	0.593	0.004

** *p* < 0.01, *** *p* < 0.001.

**Table 3:** Functional brain connectivity between brain regions.

	Brain Regions	F(df1, df2)	Greenhouse–Geisser	η^2^
Task load	Left-right prefrontal	14.533 *** (1.0, 12.3)	0.000	0.040
	Right prefrontal-occipital	12.937 *** (1.0, 11.8)	0.000	0.036
	Left-right prefrontal-occipital	0.337 (1.0, 16.5)	0.562	0.001
Time-on-task	Left-right prefrontal	47.208 *** (2.8, 29.4)	0.000	0.119
	Right prefrontal-occipital	103.475 *** (2.8, 24.6)	0.000	0.229
	Left-right prefrontal-occipital	56.694 *** (2.9, 26.2)	0.000	0.396
Task load × Time-on-task	Left-right prefrontal	17.218 *** (3.1, 39.2)	0.000	0.047
	Right prefrontal-occipital	4.254 ** (3.6, 32.8)	0.003	0.012
	Left-right prefrontal-occipital	2.665 * (3.4, 43.2)	0.032	0.030

* *p* < 0.05, ** *p* < 0.01, *** *p* < 0.001.

## Data Availability

Data will be made available upon reasonable request.
